# The association between hepatitis C virus infection status and blood pressure in adults in the United States: NHANES 1999–2012

**DOI:** 10.3389/fcimb.2024.1401323

**Published:** 2024-06-04

**Authors:** Feng Yang, Jianping Luo

**Affiliations:** Department of Cardiology, Ganzhou People’s Hospital, Ganzhou, Jiangxi, China

**Keywords:** hepatitis C virus, hypertension, diastolic blood pressure, systolic blood pressure, NHANES (National Health and Nutrition Examination Survey)

## Abstract

**Background:**

The Hepatitis C virus (HCV) infection is strongly associated with cardiovascular disease risk factors, but the relationship with blood pressure (BP) remains unclear.

**Objectives:**

To assess the association between HCV infection status and BP in US adults.

**Methods:**

Data for the study were obtained from the National Health and Nutrition Examination Survey (NHANES) between 1999 and 2012. The association of HCV infection status (including HCV infection, current HCV infection, and past HCV infection) with hypertension, systolic blood pressure (SBP), and diastolic blood pressure (DBP) were explored using logistic or linear regression analyses respectively.

**Results:**

A total of 25,850 participants (age≥18 years) were enrolled in the current study, including 14,162 participants with hypertension. After adjusting for all covariates, HCV infection/current HCV infection was not associated with hypertension and SBP compared to participants with non-HCV infection (OR: 1.34,95% CI 0.96–1.87/1.31 95% CI 0.91,1.91, β: -0.92, 95% CI -2.7–0.86/-0.35 95% CI -2.51,1.81, respectively). HCV infection/current HCV infection was only associated with elevated DBP (β: 4.1,95% CI 2.57–5.63/4.24,95% CI 2.27–6.21). However, there was no correlation with past HCV infection in participants with hypertension, SBP, and DBP compared to those with non-HCV infection (OR: 1.23,95% CI 0.59–2.54; β: -3.79, 95% CI -7.67–0.08 and 2.28 95% CI -0.36–4.92, respectively).

**Conclusion:**

In a representative sample of US adults, it was found that both HCV infection and current HCV infection were independently linked to higher DBP. However, there was no association between past HCV infection and DBP.

## Graphical Abstract

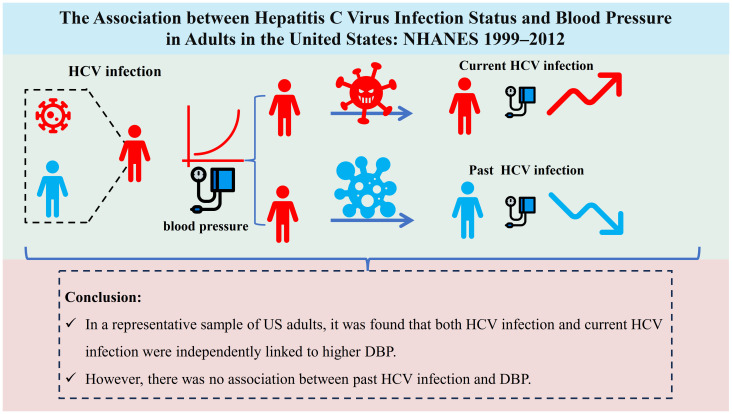

## 1 Introduction

Hepatitis C virus (HCV) infection refers to a viral hepatitis caused by an infection called HCV (Yeung et al., 2014; El-Ghitany et al., 2015), which brings about chronic inflammatory necrosis and fibrosis of the liver, and part of the patients may worsen into cirrhosis and eventually hepatocellular carcinoma (Udompap et al., 2016; Kanda et al., 2019). It is estimated that approximately 400,000 people die each year from HCV-related diseases (Pietschmann and Brown, 2019). Patients with HCV infection were often found to have hypertension, diabetes, and kidney disease, and mortality from cardiovascular disease caused by HCV infection was significantly increased (Petta et al., 2014; Lazo et al., 2017; Kuo et al., 2018; Sicras-Mainar et al., 2018). Therefore, HCV infection is closely related to cardiovascular diseases. Among US adults, hypertension is one of the most important diseases, leading to cardiovascular disease and premature death, which places a severe economic and social burden (Murray et al., 2013; Forouzanfar et al., 2017). More and more evidence shows that hypertension is one of the most common combined diseases in patients with HCV infection (Ruzicka et al., 2018; Sicras-Mainar et al., 2018; Chung et al., 2021), which has attracted the attention of clinicians in recent years.

The previous study suggested that HCV infection leads to liver damage and inflammation, which in turn may cause abnormal liver function (Sevastianos et al., 2020). The liver plays an important metabolic role in the body, including blood pressure (BP) regulation (Guzik and Touyz, 2017; Güven, 2023). Abnormal liver function may lead to dysregulation of angiotensin in the blood, which in turn may lead to increased BP (Morales et al., 2012). HCV infection may lead to functional impairment of endothelial cells in the vascular wall. Vascular endothelial cells play an important role in the regulation of BP, and their abnormal function may lead to disturbances in the regulation of vasodilation and contraction, which in turn may cause hypertension (González-Reimers et al., 2016; Konukoglu and Uzun, 2017). A study by Z M Younossi et al. showed that HCV antibody positivity was a risk factor for hypertension (Younossi et al., 2013). A recent retrospective study found that the prevalence of hypertension was significantly higher in patients with chronic HCV infection than in non-infected individuals (Butt et al., 2007). However, an analysis of the National Population Survey in Egypt found that HCV status (Past exposure and Chronic infection) was not associated with hypertension after adjusting for confounders such as BMI, smoking, age, and gender (Gadallah et al., 2018). Considering that the relationship between HCV and hypertension is controversial, more in-depth studies are needed to elucidate the correlation between HCV infection and hypertension. In addition, HCV infection status is classified as HCV antibody positive, current HCV infection, and past HCV infection, and it is unclear whether the association of different HCV infection status on BP (hypertension, systolic blood pressure [SBP], and diastolic blood pressure [DBP]) is consistent. In this study, we analyzed data from the National Health and Nutrition Examination Survey (NHANES) from 1999 to 2012 to assess the association between HCV infection and hypertension, SBP, and DBP for clinical practice.

## 2 Methods

### 2.1 Study population

The National Center for Health Statistics/Centers for Disease Control and Prevention conducted NHANES, a program to assess the health and nutritional status of adults and children in the United States. Data on NHANES were obtained by the management of standardized questionnaires and medical evaluations conducted in mobile check-up clinics, including interviews conducted at home, physical examinations conducted in mobile check-up centers, and other data, including blood collection. NHANES included non-institutionalized civilians aged over two months, with complex, multi-stage, stratified, and grouped samples. The study was mandated by the CDC Institutional Review Board and written informed consent was obtained from all participants. All participants provided written informed consent, and all methods were performed according to the relevant guidelines and regulations (NHANES, 2022).

**Figure 1:**
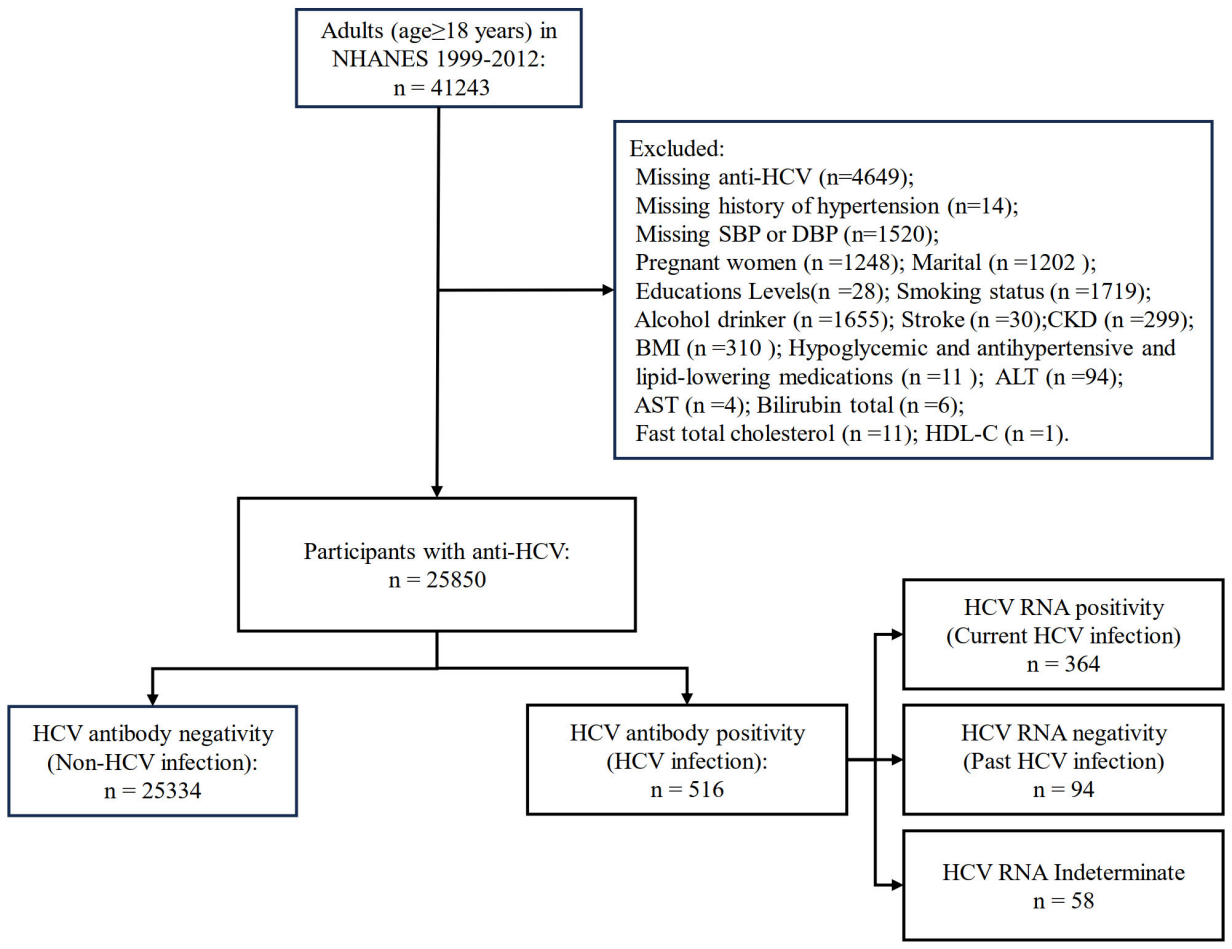
Flow diagram of inclusion criteria and exclusion criteria. NHANES, National Health and Nutrition Examination Survey; HCV, hepatitis C virus; SBP, systolic blood pressure; DBP, diastolic blood pressure; BMI, body mass index; ASCVD, atherosclerotic cardiovascular disease; CKD, chronic kidney disease; AST, aspartate aminotransferase; ALT, alanine aminotransferase; HDL-C, high-density lipoprotein cholesterol.

For this study, a total of 41,243 adults (aged ≥ 18 years) were included from NHANES 1999–2012. After excluding missing variables, 25,850 participants were finally included in this study (**Figure 1**).

### 2.2 Definition of HCV infection

NHANES performs anti-HCV screening for participants 6 years of age and older. Serum specimens will be appropriately processed, stored, and shipped frozen at -30°C to the National Centers for Disease Control and Prevention for testing (Laboratory Procedures Manual). Detailed instructions for sample collection and processing can be found on the NHANES website (NHANES, 2011–2012).

The RIBA confirmatory test was utilized to test for the presence of anti-HCV. The Chiron RIBA 3.0 Strip Immunoblot Assay, developed by Chiron Corporation, Inc., is an *in vitro* qualitative enzyme immunoassay designed to detect anti-HCV in human serum or plasma. The presence of anti-HCV reactivity in specimens is visualized by utilizing anti-human IgG enzyme conjugates in combination with a colorimetric enzyme substrate. This enables the detection of individual HCV-encoded proteins. Samples with a positive RIBA result are reported as confirmed positive for anti-HCV. Samples that yield a negative outcome in the RIBA test are documented as negative for anti-HCV, whereas samples with indeterminate RIBA results are reported as being indeterminate. RIBA-positive and indeterminate specimens were further tested for HVC RNA, and HCV RNA in human serum or plasma was quantified on a COBAS AMPLICOR analyzer to confirm the participant’s infection status (NHANES, 2013–2014).

Participants were defined as HCV infection if they were anti-HCV positive; anti-HCV positive & HCV RNA positive were defined as current HCV infection and anti-HCV positive & HCV RNA negative were defined as past HCV infection. However, some participants with anti-HCV positive from the 1999–2012 NHANES were not tested for HCV RNA, which we defined as indeterminate HCV.

### 2.3 Measurements of blood pressure

BP was evaluated during 1999–2012 using the same protocol. BP values were measured by a trained clinician using an appropriately sized BP cuff and a mercury sphygmomanometer. Get the readings after resting in a seated position for 5 minutes. Three BP measurements were obtained at 30-second intervals. The average value of all available measurements was used to define SBP and DBP levels (Ostchega et al., 2003). Hypertension was defined as SBP ≥ 130 mmHg or DBP ≥ 80 mmHg, a self-reported history of hypertension, or the use of antihypertensive medications (Muntner et al., 2018).

### 2.4 Other covariate data acquisition methods

Data was collected by managing standardized questionnaires and performing medical evaluations in mobile check-up clinics. In combination with previous studies on hypertension and chronic HCV infection and NHANES dataset variables, the following variables were collected: sex (male or female), age, race/ethnicity (Non-Hispanic White, Non-Hispanic Black, Mexican American or Other Race), education levels (below high school, college/above), marital (widowed or divorced or separated, never married, married or living with partner), alcohol drinker (never, former or now), smoking status (current smoker, former smoker or never smoker), diabetes, stroke, atherosclerotic cardiovascular disease (ASCVD), chronic kidney disease (CKD), and hyperlipidemia. Diabetes was defined as fasting blood glucose ≥126 mg/dl or glycated hemoglobin ≥ 6.5%, or the use of hypoglycemia medications (Wang et al., 2021). Estimated glomerular filtration rate (eGFR) was calculated by the Modification of Diet in Renal Disease Study equation (Levey et al., 2006). Subjects with eGFR < 60ml/min/1.73m^2^ were considered to have CKD (Murphy et al., 2016). Body mass index (BMI, calculated as weight in kilograms divided by height in meters squared), and biochemical indicators (glutamic-oxaloacetic transaminase [AST], alanine aminotransferase [ALT], alkaline phosphatase, albumin, bilirubin total, fast total cholesterol, and high-density lipoprotein cholesterol [HDL-C]).

### 2.5 Statistical analysis

Data in this study were weighted using interview sample weights provided by NHANES to account for the complexity of the NHANES database survey design (NHANES). All statistical analyses were performed using SAS 9.4 (version 9.4, SAS Institute) and R Studio software (4.2.1), and a two-sided P<0.05 was considered statistically significant.

Continuous variables are expressed by mean standard (standard error [SE]) and classified variables are expressed by percentage (%). Continuous variables were analyzed for differences between groups using the ANOVA (NHANES). Categorical variables were analyzed for differences between groups using the chi-square test. Logistic regression analysis was used to assess the association between HCV infection/current HCV infection and past HCV infection and hypertension. Results were expressed as odds ratios (ORs) and 95% confidence intervals (95% CIs). Linear regression analysis was used to assess the association between HCV infection/current HCV infection and past HCV infection and DBP, and SBP. Results were presented as standardized coefficients (β) and 95% CIs. We constructed three models (Model 1 was adjusted for age, and sex. Model 2 was adjusted for age, sex, race, and education levels, marital, smoking status, alcohol drinker. Model 3 was adjusted for the variables in model 2 plus hypoglycemic medications, antihypertensive medications, lipid-lowering medications, diabetes, stroke, ASCVD, CKD, BMI, ALT, AST, albumin, bilirubin total, alkaline phosphatase, fast total cholesterol, and HDL-C. We also examined the association between HCV infection status and DBP by stratifying analyses by age (≤65 and >65 years), race/ethnicity (Non-Hispanic white, Non-Hispanic black, Mexican American, and Other Race), education levels (below high school and college/above), smoking status, alcohol drinker, and BMI (≤25 and >25kg/m^2^), and stratification interactions were tested.

## 3 Results

### 3.1 Baseline characteristics of participants with different HCV infection status

A total of 25,850 participants were enrolled in the current study, including 516 individuals infected with hepatitis C (current infection: 364, past infection: 94, and HCV RNA undetermined: 58). The age of HCV infection, current HCV infection and past HCV infection was 47.93, 48.09 and 47.42, respectively. The participants with HCV infection were more likely to be men, had more education, were more likely to be smokers, obesity and stroke, had higher levels of alkaline phosphatase, ALT, and AST, and lower levels of fast total cholesterol. Compared with those with current HCV infection, past HCV infection were more likely to be female, have stroke and hyperlipidemia, and have lower ALT, AST, alkaline phosphatase, and higher fast total cholesterol (**Table 1**).

**Table 1:** The participant baseline characteristics by HCV infection status among US adults in NHANES (1999–2012).

Character	Total	Anti-Hepatitis C (confirmed), N (weighted%)					*P-*value Positive/ Negative	*P-*value Current/Past
		Negative	Positive					
			All	Indeterminate	Current	Past		
No. of participants in sample	25850	25334	516	58	364	94		
Age (years), mean (SE)	46.29 (0.24)	46.28 (0.24)	47.18 (0.58)	48.02 (1.29)	47.51 (0.56)	45.67 (1.70)	0.15	0.29
**Sex**							< 0.01	0.01
Male	13217 (49.69)	12879 (49.39)	338 (66.15)	37 (70.82)	242 (69.14)	59 (54.09)		
Female	12633 (50.31)	12455 (50.61)	178 (33.85)	21 (29.18)	122 (30.86)	35 (45.91)		
**Race/ethnicity**							< 0.01	0.02
Non-Hispanic white	12960 (73.00)	12740 (73.08)	220 (68.57)	19 (60.39)	146 (65.76)	55 (81.95)		
Non-Hispanic black	5030 (9.75)	4855 (9.59)	175 (18.34)	13 (11.74)	145 (22.37)	17 (9.28)		
Mexican American	4726 (7.16)	4650 (7.17)	76 (6.59)	19 (11.95)	44 (6.20)	13 (4.87)		
Other Race	3134 (10.09)	3089 (10.16)	45 (6.50)	7 (15.92)	29 (5.67)	9 (3.90)		
**Educations Levels**							< 0.01	0.89
Below high school	7199 (18.10)	7010 (17.90)	189 (28.61)	30 (41.67)	130 (26.56)	29 (27.84)		
College/Above	18651 (81.90)	18324 (82.10)	327 (71.39)	28 (58.33)	234 (73.44)	65 (72.16)		
**Smoking status**							< 0.01	0.49
Never	13407 (51.50)	13323 (52.17)	84 (15.66)	12 (16.14)	59 (16.39)	13 (13.10)		
Former	6759 (25.09)	6624 (25.14)	135 (22.66)	18 (28.56)	87 (20.29)	30 (26.88)		
Now	5684 (23.41)	5387 (22.70)	297 (61.68)	28 (55.30)	218 (63.32)	51 (60.02)		
**Alcohol drinker**							< 0.01	0.25
Never	3477 (11.24)	3455 (11.36)	22 (5.15)	2 (2.10)	14 (4.03)	6 (10.37)		
Former	5081 (16.16)	4916 (16.03)	165 (23.52)	20 (16.99)	115 (25.08)	30 (22.18)		
Now	17292 (72.59)	16963 (72.62)	329 (71.33)	36 (80.91)	235 (70.88)	58 (67.45)		
**Marital**							< 0.01	0.64
Widowed or divorced or separated	5770 (18.11)	5612 (17.95)	158 (27.11)	19 (43.24)	115 (26.38)	24 (20.49)		
Never married	4263 (16.88)	4170 (16.86)	93 (18.10)	10 (14.87)	62 (18.08)	21 (19.98)		
Married or living with partner	15817 (65.01)	15552 (65.20)	265 (54.79)	29 (41.89)	187 (55.54)	49 (59.53)		
Poverty, mean (SE)	3.05 (0.04)	3.07 (0.04)	2.15 (0.10)	2.24 (0.31)	2.08 (0.12)	2.33 (0.21)	< 0.01	0.28
SBP, mean (SE), mmHg	122.44 (0.23)	122.42 (0.24)	123.20 (1.00)	126.79 (2.83)	123.93 (1.19)	118.91 (2.41)	0.45	0.07
DBP, mean (SE), mmHg	71.53 (0.21)	71.47 (0.21)	74.84 (0.77)	78.11 (1.44)	74.84 (0.95)	73.03 (1.50)	< 0.01	0.29
BMI, mean (SE), kg/m^2^	28.33 (0.08)	28.35 (0.08)	27.18 (0.33)	27.61 (0.95)	26.97 (0.48)	27.59 (0.62)	< 0.01	0.47
Medications Used								
Hypoglycemic	2485 (6.14)	2432 (6.16)	53 (5.07)	7 (5.25)	39 (5.17)	7 (4.64)	0.28	0.84
Antihypertensive	7805 (24.05)	7642 (24.03)	163 (25.20)	20 (23.35)	118 (26.54)	25 (21.97)	0.62	0.45
Lipid-lowering	4378 (13.56)	4339 (13.72)	39 (5.20)	4 (5.01)	23 (4.80)	12 (6.56)	< 0.01	0.45
Baseline comorbidities								
Hypertension	14162 (49.74)	13836 (49.57)	326 (58.80)	40 (65.92)	237 (59.04)	49 (54.09)	0.01	0.58
Diabetes	3017 (7.64)	2952 (7.65)	65 (7.20)	10 (6.50)	46 (7.79)	9 (5.69)	0.74	0.54
Stroke	967 (2.55)	935 (2.52)	32 (4.18)	3 (3.72)	19 (2.28)	10 (10.45)	0.04	< 0.01
ASCVD	2650 (7.63)	2593 (7.61)	57 (8.67)	10 (13.26)	34 (6.10)	13 (14.24)	0.48	0.06
CKD	4714 (13.37)	4612 (13.33)	102 (15.24)	11 (10.26)	74 (14.23)	17 (21.19)	0.33	0.3
COPD	1224 (4.03)	1169 (3.95)	55 (8.18)	6 (7.62)	35 (7.93)	14 (9.31)	< 0.01	0.65
Hyperlipidemia	19044 (72.87)	18722 (73.01)	322 (65.53)	35 (65.80)	209 (57.96)	78 (89.30)	0.01	< 0.01
Biochemical tests, mean (SE)								
ALT, U/L	26.23 (0.24)	25.63 (0.23)	58.03 (3.51)	78.81 (13.31)	64.68 (4.43)	25.52 (1.53)	< 0.01	< 0.01
AST, U/L	25.38 (0.14)	24.89 (0.12)	51.48 (2.61)	64.59 (8.02)	56.27 (3.25)	29.08 (3.39)	< 0.01	< 0.01
Albumin, g/dl	4.32 (0.01)	4.33 (0.01)	4.19 (0.03)	4.27 (0.10)	4.17 (0.03)	4.22 (0.04)	< 0.01	0.26
Bilirubin total, mg/dl	0.73 (0.01)	0.73 (0.01)	0.76 (0.02)	0.68 (0.04)	0.80 (0.03)	0.69 (0.03)	0.05	0.01
Alkaline phosphatase, u/L	69.81 (0.47)	69.63 (0.46)	79.70 (2.30)	87.85 (6.82)	80.75 (2.63)	71.89 (3.10)	< 0.01	0.02
Fast total cholesterol, mg/dl	199.72 (0.51)	199.92 (0.51)	188.58 (2.34)	186.54 (6.50)	182.16 (2.28)	209.99 (6.36)	< 0.01	< 0.01
HDL cholesterol, mg/dl	52.19 (0.21)	52.18 (0.22)	52.82 (1.21)	51.83 (4.86)	54.18 (1.23)	49.03 (2.37)	0.62	0.05

Numbers (n) in the table were unweighted.

Percentages or means (SE) were estimated using US population weights.

Race/ethnicity was determined using preferred terminology from the National Center for Health Statistics as non-Hispanic white, non-Hispanic black, and Mexican American. Mexican-American individuals were oversampled rather than broader groups of individuals from Latin America. Other include Asian, other Hispanic, Alaskan native, and multiracial individuals.

N, number; HCV, hepatitis C virus; BMI, body mass index; ASCVD, atherosclerotic cardiovascular disease; CKD, chronic kidney disease; AST, aspartate aminotransferase; ALT, alanine aminotransferase; HDL-C, high-density lipoprotein cholesterol; SBP, systolic blood pressure; DBP, diastolic blood pressure.

### 3.2 The association between HCV infection status and hypertension, SBP and DBP

We first assessed the effect of HCV infection on hypertension (**Table 2**). After adjusting for age, and sex (Model 1), HCV infection was not associated with hypertension and SBP compared with non-HCV participants (OR: 1.25, 95% CI 0.92–1.7 and β: -0.67, 95% CI -2.57–1.24). After adjusting for age, sex, race, and education levels, marital, smoking status, alcohol drinker, hypoglycemic medications, antihypertensive medications, lipid-lowering medications, diabetes, stroke, ASCVD, CKD, BMI, ALT, AST, albumin, bilirubin total, alkaline phosphatase, fast total cholesterol, and HDL-C (Model 3), HCV infection remained unrelated to hypertension and SBP (OR: 1.34, 95% CI 0.96–1.87 and β: -0.92, 95% CI -2.7–0.86). However, HCV infection was associated with increased DBP compared to non-HCV participants (β: 4.1,95% CI 2.57–5.63) in Model 3).

**Table 2:** The association between HCV infection state and hypertension, systolic blood pressure and diastolic blood pressure among US adults in NHANES (1999–2012).

Character	HCV infection		Current HCV infection		Past HCV infection	
	OR (95%CI)	*P* value	OR (95%CI)	*P* value	OR (95%CI)	*P* value
Hypertension						
** Model 1**	1.25 (0.92,1.7)	0.15	1.19 (0.86,1.66)	0.29	1.22 (0.62,2.42)	0.56
** Model 2**	1.25 (0.91,1.72)	0.16	1.2 (0.86,1.69)	0.29	1.21 (0.61,2.41)	0.58
** Model 3**	1.34 (0.96,1.87)	0.08	1.31 (0.91,1.91)	0.15	1.23 (0.59,2.54)	0.58
Blood pressure	*β* (95%CI)	*P* value	*β* (95%CI)	*P* value	*β* (95%CI)	*P* value
Systolic blood pressure						
** Model 1**	-0.67 (-2.57,1.24)	0.49	-0.4 (-2.65,1.84)	0.72	-3.22 (-7.04,0.61)	0.1
** Model 2**	-0.88 (-2.81,1.05)	0.37	-0.53 (-2.86,1.8)	0.65	-3.56 (-7.38,0.25)	0.07
** Model 3**	-0.92 (-2.7,0.86)	0.31	-0.35 (-2.51,1.81)	0.75	-3.79 (-7.67,0.08)	0.054
Diastolic blood pressure						
** Model 1**	2.82 (1.19,4.46)	**<0.01**	2.69 (0.67,4.72)	**0.01**	1.4 (-1.53,4.33)	0.34
** Model 2**	3.56 (1.94,5.18)	**<0.01**	3.38 (1.39,5.38)	**<0.01**	2.26 (-0.74,5.25)	0.14
** Model 3**	4.1 (2.57,5.63)	**<0.01**	4.24 (2.27,6.21)	**<0.01**	2.28 (-0.36,4.92)	0.09

Model 1 was adjusted for age, sex, race/ethnicity.

Model 2 was adjusted for age, sex, race/ethnicity, and education levels, poverty, marital, smoking status, alcohol drinker.

Model 3 was adjusted for the variables in model 2 plus hypoglycemic medications, antihypertensive medications, lipid-lowering medications, diabetes, stroke, ASCVD, CKD, hyperlipidemia, BMI, ALT, AST, albumin, bilirubin total, alkaline phosphatase, fast total cholesterol, HDL-C.

NHANES, National Health and Nutrition Examination Survey; HCV, hepatitis C virus; BMI, body mass index; ASCVD, atherosclerotic cardiovascular disease; CKD, chronic kidney disease; AST, aspartate aminotransferase; ALT, alanine aminotransferase; HDL-C, high-density lipoprotein cholesterol; OR, odds ratio; CI, confidence interval. Bold font: P<0.05.

The association between current and past HCV infection and BP was also assessed (**Table 2**), A similar relationship was observed. HCV current infection was not associated with hypertension and SBP compared with non-HCV participants, with OR: 1.31 (95% CI 0.91,1.91) and β: -0.35 (95% CI -2.51,1.81) in Model 3, respectively. DBP was higher in HCV current infection (β: 4.24,95% CI 2.27–6.21 in Model 3). Interestingly, there was no association with past HCV infection in participants with hypertension, SBP, and DBP compared to those with non-HCV infection (OR: 1.23,95% CI 0.59–2.54; β: -3.79, 95% CI -7.67–0.08 and 2.28 95% CI -0.36–4.92, respectively).

### 3.3 Stratified analysis

**Figure 2:**
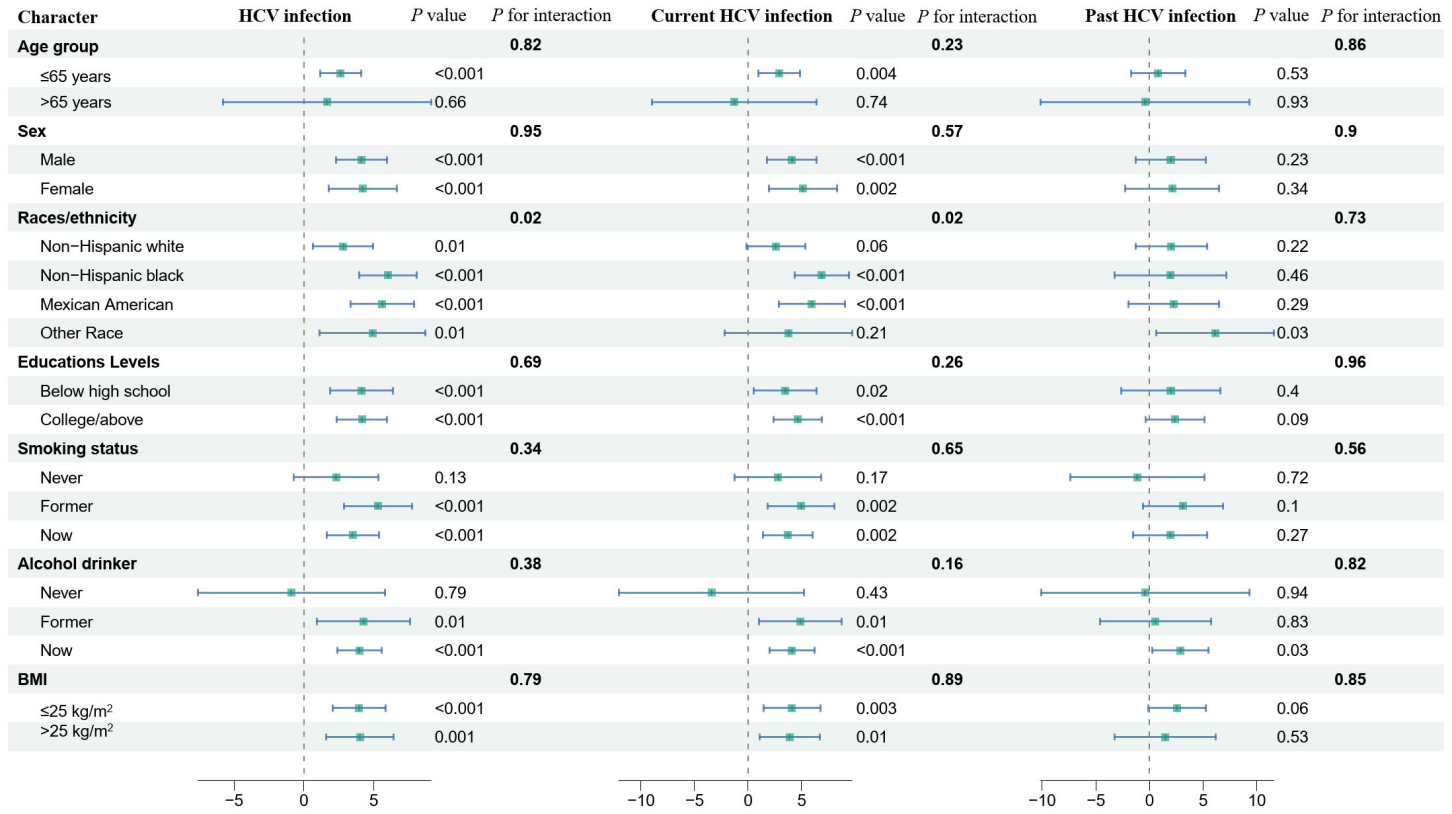
Association between HCV infection and hypertension, systolic blood pressure, and diastolic blood pressure exposure in stratified analyses. The model was adjusted for age, sex, race/ethnicity, and education levels, marital, smoking status, alcohol drinker, hypoglycemic medications, antihypertensive medications, lipid-lowering medications, diabetes, stroke, ASCVD, CKD, hyperlipidemia, BMI, ALT, AST, albumin, bilirubin total, alkaline phosphatase, fast total cholesterol, HDL-C. NHANES, National Health and Nutrition Examination Survey; HCV, hepatitis C virus; SBP, systolic blood pressure; DBP, diastolic blood pressure; BMI, body mass index; ASCVD, atherosclerotic cardiovascular disease; CKD, chronic kidney disease; AST, aspartate aminotransferase; ALT, alanine aminotransferase; HDL-C, high-density lipoprotein cholesterol; OR, odds ratio; CI, confidence interval.

Stratified analyses were performed for age (≤65 and >65 years), races/ethnicity (Non-Hispanic white, Non-Hispanic black, Mexican American, Other Race), education levels (below high school, college/above), smoking status, alcohol drinker, and BMI (≤25kg/m^2^, >25kg/m^2^) as shown in **Figure 2**. Results indicated heterogeneity in the association between HCV infection status and DBP between Races/ethnicity. The association between HCV infection and current HCV infection and DBP was stronger in non-Hispanic blacks. No association between current HCV infection and DBP was observed in Non-Hispanic white or Other Race.

## 4 Discussion

In this large cross-sectional study, we investigated the association between HCV infection status and BP in US adults. Our study found that HCV infection (anti-HCV positive) and current HCV infection were associated with increased DBP but not with hypertension or SBP among US adults, after adjusting for important potential confounders. However, past HCV infection was not associated with hypertension, SBP, or DBP. Our study suggests that HCV infection increases DBP, but clearance of the virus reversed the association.

Our study showed that HCV infection was not associated with hypertension. This conclusion is supported by the findings of Gadallah M (Gadallah et al., 2018). However, another study by Z.M. Younososi et al. came to the opposite conclusion (Chronic HCV patients have a 106% increased risk of hypertension), which may be related to the different adjusted variables (Younossi et al., 2013). The study only adjusted for the variables of age, race, and obesity. However, other confounding factors, such as total bilirubin, ALT, alkaline phosphatase, and AST may also affect blood pressure. Studies have shown that total bilirubin, ALT, alkaline phosphatase, and AST in people with HCV infection are significantly different from those without HCV infection (Arase et al., 2003; Eminler et al., 2014) and that these factors are also associated with BP (Ramsay, 1977; Kunutsor et al., 2017; Kuchulakanti et al., 2020). Our study added these confounders to make the results more reliable. In the study, we also found a correlation between HCV infection and DBP. The mechanism of correlation between HCV infection and DBP is currently unknown. HCV infection can also cause hepatic necroinflammation, atherosclerosis, and vascular cirrhosis (Di Pietro et al., 2017; Sevastianos et al., 2020). This metabolic change may affect blood pressure by altering the function and structure of blood vessels (Wang et al., 2024). In addition, a cross-sectional study showed that HCV-infected individuals had a higher 24-hour mean heart rate than non-HCV infection (Poliwczak et al., 2020). Higher heart rate, arteriosclerosis, and vascular sclerosis have been associated with increased DBP (Koskela et al., 2013; Wen et al., 2015).

Although some studies have suggested that some of the drugs used to treat HCV may lead to increased BP (Gamal Eldeen et al., 2022). However, the results of our study found no difference in BP (hypertension, SBP, and DBP) between those with past HCV infection and non-HCV infection. This one is an important finding. DBP elevation can be improved after virus clearance in HCV patients. Previous studies have shown that the prevalence of hypertension is reduced in patients with antiviral therapy compared to patients with HCV infection who do not use antiviral therapy (Carrat et al., 2019). The following possibilities exist for the potential mechanisms by which hepatitis C patients can reduce DBP after clearance of the virus. Firstly, one possible reason to consider is the reduction of HCV-induced liver inflammation and fibrosis after HCV antiviral therapy, which improves liver function (Shigefuku et al., 2016). Healthy liver function is essential for maintaining normal BP, as it is involved in the regulation of water and salt balance in the kidneys and in the release of some important *in vivo* regulators such as angiotensin (Henriksen et al., 2006; Møller and Henriksen, 2008). In addition, the treatment of HCV usually requires patients to follow a healthy lifestyle that includes abstaining from alcohol and smoking, eating a balanced diet, and exercising in moderation (Nobili et al., 2011; Sublette et al., 2013). These lifestyle changes may lead to an improvement in the patient’s overall health, thereby reducing the risk of elevated BP (Samadian et al., 2016; Valenzuela et al., 2021).

The results of this study have important implications. Firstly, the health benefits of clearing the HCV for hepatitis C patients were confirmed, especially the reduction of DBP. Secondly, in patients with hypertension, and especially co-infected with HCV, the treatment of HCV may have a positive impact on BP management and mitigate their cardiovascular risk. Moreover, this result also highlights the important role of the liver in regulating BP, furthering the understanding of the interrelationship between the liver and the circulatory system.

There are many strengths in this study. Strengths of the current study include a prospective study design, a relatively large sample size, and the use of a nationally representative sample of adults in the US, which helps in the generalizability of our findings. Despite this, there are limitations to our study. Although we were able to adjust for the major risk factors identified in the previous studies for HCV infection and hypertension, the possibility of residual confounding cannot be ruled out. A small proportion of HCV antibody-positive participants were not tested for HCV RNA, and this may have had an impact on our assessment of the association between current HCV and past HCV infection and BP. The NHANES does not sample institutionalized or homeless individuals, who are expected to have higher HCV prevalence. This research is a cross-sectional study, so a causal association between elevated DBP and HCV infection was not demonstrated.

### 4.1 Conclusions

In conclusion, we found a correlation between HCV infection and current HCV infection with increased DBP, but not with hypertension and SBP in US adults. Hypertension, as a major co-morbidity in HCV-infected patients (Sicras-Mainar et al., 2018), contributes to rising costs for the healthcare system and society. Our findings provide a basis for education and interventions to prevent and better control hypertension.

## Data availability statement

Publicly available datasets were analyzed in this study. This data can be found here: Research data can be obtained from https://www.cdc.gov/nchs/nhanes/.

## Ethics statement

The studies involving humans were approved by This study does not contain any studies with human participants or animals performed by any of the authors. This study only involved secondary data analysis of the existing NHANES database, which is publicly available and has been de-identified, thus there is no need to apply an IRB approval from our institution.. The studies were conducted in accordance with the local legislation and institutional requirements. The participants provided their written informed consent to participate in this study.

## Author contributions

FY: Data curation, Formal Analysis, Investigation, Methodology, Software, Visualization, Writing – original draft. JL: Data curation, Investigation, Methodology, Software, Supervision, Validation, Visualization, Writing – review & editing.
